# False-Negative Rate of Gram-Stain Microscopy for Diagnosis of Septic Arthritis: Suggestions for Improvement

**DOI:** 10.1155/2014/830857

**Published:** 2014-02-13

**Authors:** Paul Stirling, Radwane Faroug, Suheil Amanat, Abdulkhaled Ahmed, Malcolm Armstrong, Pankaj Sharma, Ahmed Qamruddin

**Affiliations:** ^1^Medical School, University of Manchester, Stopford Building, Oxford Road, Manchester M13 9PT, UK; ^2^Department of Orthopaedic Surgery, Manchester Royal Infirmary, Manchester M13 9WL, UK; ^3^Department of Orthopaedic Surgery, Royal Preston Hospital, Preston PR2 9HT, UK; ^4^Department of Microbiology, Manchester Royal Infirmary, Manchester M13 9WL, UK

## Abstract

We quantify the false-negative diagnostic rate of septic arthritis using Gram-stain microscopy of synovial fluid and compare this to values reported in the peer-reviewed literature. We propose a method of improving the diagnostic value of Gram-stain microscopy using Lithium Heparin containers that prevent synovial fluid coagulation. Retrospective study of the Manchester Royal Infirmary microbiology database of patients undergoing synovial fluid Gram-stain and culture between December 2003 and March 2012 was undertaken. The initial cohort of 1896 synovial fluid analyses for suspected septic arthritis was reduced to 143 after exclusion criteria were applied. Analysis of our Gram-stain microscopy yielded 111 false-negative results from a cohort size of 143 positive synovial fluid cultures, giving a false-negative rate of 78%. We report a false-negative rate of Gram-stain microscopy for septic arthritis of 78%. Clinicians should therefore avoid the investigation until a statistically significant data set confirms its efficacy. The investigation's value could be improved by using Lithium Heparin containers to collect homogenous synovial fluid samples. Ongoing research aims to establish how much this could reduce the false-negative rate.

## 1. Introduction

We find that synovial fluid Gram-stain microscopy as used at present is of no value in the diagnosis of septic arthritis, with a false negative diagnosis rate of 78%, as we show by analysing microbiological data collected during 9 years at the Manchester Royal Infirmary.

Our result is consistent with previous values reported in the literature, which show a false-negative rate of 25–50% [1–3], and highlights the need to educate clinicians that the diagnosis of septic arthritis should be based on clinical grounds and without reliance on synovial fluid Gram-stain microscopy. Although synovial fluid culture is considered the gold standard, its practical application is also limited by the 24-hour time delay before preliminary results are available, and the additional 24 hours or more needed before final results can be known. This is ill-suited to the diagnosis of septic arthritis, which is an orthopaedic emergency with a significant morbidity and mortality rate [4, 5].

Alternative diagnostic techniques remain underexplored, but a proposed modification to existing techniques is the use of Lithium Heparin containers to collect the synovial fluid. By preventing coagulation of the fluid, Lithium Heparin tubes can produce more homogenous samples for microscopic examination. Research is now being undertaken to quantify how much this modification would reduce the false-negative rate of synovial fluid Gram-stain microscopy in the clinical diagnosis of septic arthritis.

## 2. Aims

1. To quantify the false negative rate of Gram-stain microscopy of synovial fluid in diagnosing septic arthritis at the Manchester Royal Infirmary compared with values reported in the peer-reviewed literature.
2. To identify means for improving the diagnostic value of Gram-stain microscopy for the diagnosis of septic arthritis.

## 3. Standard 

The false negative rate for Gram-stain microscopy reported in the literature is 25–50% [1–3]. In the absence of formal guidelines from The Royal College of Surgeons of England, The Royal College of Surgeons of Edinburgh, The British Orthopaedic Association, or The British Society for Rheumatology, this range was accepted as the standard for this audit.

## 4. Methods 

A search of the Manchester Royal Infirmary (MRI) microbiology database for all patients undergoing synovial fluid culture between December 2003 and March 2012 was performed. During this period, the microbiology investigation method remained unchanged: Gram-stain microscopy and microbial culture on direct plates (for 48 hours) and in enrichment broth (for up to 5 days) were performed after centrifugation of synovial fluid received in a sterile sample pot.

This study assumed that synovial fluid culture is the gold standard for diagnosis of septic arthritis; therefore only patients with synovial fluid culture positive for growth of microorganisms were included. Positive cultures from both native and prosthetic joints were included. Care was taken to eliminate cultures that could have arisen due to contamination of samples. The following exclusion criterion was defined: patients with synovial fluid cultures grown from Brain-Heart-Infusion enrichment broth (BHI) or from direct culture which grew coagulase-negative Staphylococci, Diphtheroids, alpha-haemolytic Streptococci, or fungi were excluded unless successive cultures in these patients corroborated these results. Multiple investigations on the same patient were included as the purpose of this study was to correlate Gram-stain microscopy and synovial fluid culture. Correlation of these results allowed calculation of the false negative rate of Gram-stain microscopy.

A positive microscopy result was the one that was reported as positive for presence of microorganisms in the Gram film, while a negative result reported absence of microorganisms on microscopy. As the inclusion criterion was a positive synovial fluid culture, a false negative result was defined as a Gram-stain microscopy report negative for presence of corroborating microorganisms.

## 5. Results

### 5.1. Cohort

Retrospective analysis of the microbiology database yielded 1896 synovial fluid analyses between December 2003 and March 2012. Of these, only 295 (15.5%) were positive cultures. Of the 295 positive cultures, 146 results were excluded according to the exclusion criteria outlined in the methods section. Gram-stain microscopy reports were unavailable in 6 cases of the remaining 149 results and these were excluded as correlation of the two tests was not possible. This gave a final cohort size of *n* = 143 positive synovial fluid culture results.

### 5.2. Calculation of the False Negative Rate

Of 143 positive synovial fluid cultures, Gram-stain microscopy was negative in 111 cases. This gives a false negative rate of 78% for Gram-stain microscopy.

## 6. Discussion

### 6.1. Gram-Stain Microscopy of Synovial Fluid

Septic arthritis is an orthopaedic emergency. Early diagnosis and intervention is essential to limit the associated morbidity and mortality. Delayed treatment of septic arthritis can lead to rapid destruction of the joint articular cartilage resulting in pain, stiffness, loss of function, overwhelming sepsis, and death. Reports in the literature suggest case-fatality to be as high as 11% [4]. Irreversible loss of joint function is another serious complication with rates reportedly between 25 and 50% [5–7].

Joint aspiration for synovial fluid analysis and culture is the standard investigation in suspected septic arthritis. Aspirates are sent for Gram-stain microscopy, culture, and polarised light microscopy; the latter of which can diagnose the crystal arthropathies gout and pseudogout, important differential diagnoses in suspected septic arthritis. Gram-stain microscopy of synovial fluid can rapidly confirm a diagnosis of septic arthritis but carries an inherent false negative rate between 25 and 50% [1–3]. Our study gave a higher false-negative rate of 78% for Gram-stain microscopy. It is important to note that only 15.5% of all synovial fluid analysed in our department over this period cultured positive for bacteria. This is consistent with prevalence rates for septic arthritis in patients presenting with acute monoarthritis which are reported in the literature as 8–27% [8, 9], suggesting that septic arthritis is far less common than other differential diagnoses such as the crystal arthropathies.

An extensive literature review published by Swan et al. in 2002 [10] highlighted serious shortcomings in understanding, research and quality control of synovial fluid analysis for septic arthritis. The review identified only one paper published by Shmerling in 1994 [1] which had undertaken statistical analysis to determine the sensitivity and specificity of Gram-stain microscopy and synovial fluid culture. This paper reported sensitivity of 50–75% and specificity of “quite high” for Gram-stain microscopy and sensitivity of 75–95% and specificity of >90% for culture. Assessing this paper, it is hard to comment on these statistical calculations. Shmerling's methodology is unclear and it could be that the data required for calculation of these values came from an earlier study which prospectively analysed 100 patients undergoing aspiration for various reasons and retrospectively analysed 19 patients with confirmed septic arthritis [8]. Since these publications, little has been reported to confirm or refute these results. Gram stain microscopy is widely advocated as an important adjunct in clinical diagnosis; however no authors have directly challenged the original results. 

The significant morbidity and mortality associated with septic arthritis demand a rapid and accurate investigation capable of confirming or negating the diagnosis. The high sensitivity (75–95%) of synovial fluid culture makes it a useful investigation for confirmation of septic arthritis, but as culture takes 24–48 hours or more to complete, its usefulness in directing initial management is limited. At the time of writing the only paper analysing the sensitivity and specificity of Gram-stain microscopy was published by Shmerling in 1994 [1]. A recent systematic review into management of septic arthritis conducted by Mathews et al. in 2007 analysed 80 papers but could not make any definitive recommendations for diagnosis of septic arthritis [11]. The main conclusion of this review was that not enough good-quality evidence exists to make recommendations for the best diagnostic tests in septic arthritis. 

With Gram-stain microscopy still relied upon in the diagnosis of septic arthritis, it is striking that in the intervening 20 years since Schmerling's research to determine the accuracy of this test very few further studies have since challenged its diagnostic value or suggested improved alternatives. Regardless of the initial accuracy of Shmerling's findings, Gram-stain microscopy in our department has a false negative rate of 78% for diagnosis of septic arthritis, significantly higher than that reported in the literature (25–50%). One explanation for this could be that the actual false-negative rate for Gram-stain microscopy is higher than that previously documented by Shmerling, and this calls into questioning the value of the test as a diagnostic tool.

It is worth emphasising that the diagnosis of septic arthritis remains first and foremost clinical, and “patients with a short history of a hot swollen and tender joint with restriction of movement should be regarded as having septic arthritis until proven otherwise” [4].

An important limitation of our study is that our results were not correlated clinically. This study assumed that synovial fluid culture is the gold standard for diagnosis of septic arthritis despite the fact that this investigation carries an inherent false-negative rate of 5–25% [1]. It is possible that patients with septic arthritis were not included in our study due to false-negative culture reports. This aspect of the study could be improved in future by correlation of clinical diagnosis with microbiological findings

### 6.2. Improving the False-Negative Rate of Gram-Stain Microscopy in Septic Arthritis

Good aseptic technique will reduce the incidence of sample contamination, but the main target area to improve sensitivity of Gram-stain microscopy should be the method used to collect and process the synovial fluid.

In our department, synovial fluid is currently sent for Gram-stain, microscopy, and culture in a Sterilin sterile pot. Although synovial fluid under normal circumstances contains no clotting factors, acute inflammatory conditions such as septic arthritis, osteoarthritis, or crystal arthropathies can cause local soft tissue swelling. As a result of increased local vascular permeability, clotting factors can become present in synovial fluid, causing coagulation of the fluid in the container before it has reached the lab for analysis [12]. Due to the resulting gelatinous nature of the fluid it is extremely difficult to prepare an adequate smear from the sample for effective microscopy [13]. The goal therefore should be to collect synovial fluid for Gram-stain microscopy in such a way as to prevent coagulation. Preventing coagulation of the synovial fluid sample allows more effective centrifugation using a cytospin centrifuge, which in turn leads to preparation of a more homogenous slide for analysis by microscopy. We believe this will improve the false negative Gram-stain rate in our department.

Research into varying methods of synovial fluid collection already exists. Brannan and Jerrard recommended the use of tubes containing EDTA anticoagulant for collection of synovial fluid for microscopy [14]. The use of EDTA tubes is fundamentally limited by the calcium-sequestering properties of EDTA, which dissolves calcium pyrophosphate crystals so making the diagnosis of pseudogout by crystal analysis of the same sample impossible [12]. Although this problem is surmountable by the collection of synovial fluid in several different containers, this solution is inefficient in the clinical setting. Heparin is another potential anticoagulant which is widely available. Its use in preventing coagulation of synovial fluid has been proposed since 1998 [15]. The use of 2 mL containers with Lithium Heparin was further advocated by Denton in a recent review [12]. Lithium Heparin does not interfere with endogenous crystals and so it will allow crystal analysis and microscopy to be conducted on the same sample. In addition, Lithium Heparin does not crystallise and so it will not produce false-positive results for crystal analysis [12].

In making recommendations for collection of synovial fluid, ease of use must also be taken into account. It is for this reason that we suggest the use of a 2.0 mL polystyrene Lithium Heparin bottle such as the one marketed by Teklab for microscopy and for crystal analysis. The Lithium Heparin bottle we suggest using is manufactured aseptically and the Lithium Heparin solution is sterile; however the product is not assembled in a formally sterile environment. The manufacturers are able to sterilise the product after assembly but this does not rule out the small possibility of contamination of the product during assembly, which could result in false-positive Gram-stain microscopy results. An end-sterilised Lithium heparin container in any event would be advisable, so as to avoid inadvertent cross-contamination to any synovial fluid subsequently deposited into another container for culture, if the arthrocentesis syringe needle is not changed between samples. Changing needles on syringes is best avoided, due to the risk of needlestick injury, and this step would not be required if a sterile Lithium heparin bottle is used. For synovial fluid culture, there is evidence available proposing direct collection into blood culture bottles, thus reducing manipulation of the sample and so reducing risk of contamination [16–18]. Moreover direct inoculation into blood culture bottles increases the diagnostic yield of synovial fluid culture [16, 17]. This method is applicable to both natural and prosthetic joint infections [17, 18]. For analysis of synovial fluid samples in patients with suspected septic arthritis we therefore recommend sending 1-2 mls of fluid in a Lithium Heparin container for Gram-stain and for polarised-light microscopy as proposed by Denton [12] and inoculation of fluid directly into aerobic and anaerobic blood culture bottles.

## 7. Recommendations

1. Arthrocentesis should be performed using aseptic technique.
2. Synovial fluid samples for Gram-stain microscopy should be sent in a sterile Lithium Heparin container.
3. For culture, synovial fluid should be inoculated into suitable blood culture bottles, taking care to avoid introducing contamination during this step.

We suggest evaluation of these recommendations in our Orthopaedics, Rheumatology, and Accident and Emergency Departments. A prospective audit will be undertaken to ascertain whether this reduces the false-negative rate for Gram-stain microscopy in septic arthritis.
